# The Multifaceted Role of Corridors in Residential Care Facilities: A Phenomenological Hermeneutic Study With Older Adults

**DOI:** 10.1177/19375867261431866

**Published:** 2026-04-10

**Authors:** Karin Elias, Qarin Lood

**Affiliations:** 1 The Adminstration of Eldercare and Welfare, City of Gothenburg, Gothenburg, Sweden; 2 Department of Health and Rehabilitation, Institute of Neuroscience and Physiology, 70712 The Sahlgrenska Academy, University of Gothenburg, Gothenburg, Sweden

**Keywords:** built environment, long-term care facilities, patient-centered care, person-centered care, environment of care, hallways

## Abstract

**Aim:**

The aim of this study was to explore meanings embedded in corridors and how these relate to older adults’ experiences of physical movement and their living environment within a residential care facility.

**Background:**

Residential care facilities should serve as both care environments and homes. Nevertheless, many facilities are designed like hospitals, prioritizing care over a homelike environment. Corridors are a common feature in residential care facilities’ communal areas, yet little is known about older adults’ experiences of these spaces.

**Methods:**

The study had an explorative qualitative design, adopting a phenomenological hermeneutic method. Three men and three women between 81 and 96 years of age were interviewed when moving through residential care facility corridors. Two men and two women participated one follow-up interview each.

**Results:**

The interpretation led to the overarching theme *Corridors’ multifaceted role in shaping lived spaces*. This theme comprises seven sub-themes which portray meanings ranging from physical aspects through to emotional impact, incentives, integrity, habituation, symbolic meanings of institutions and power, and how corridors may lack in meeting personal needs. Themes were further interpreted in relation to theories on sociomateriality, place/placelessness, and affordances.

**Conclusions:**

The findings highlight corridors as material and symbolic spaces that influence older adults’ well-being. However, there is also a risk of existential placelessness when personal connection with the environment is lacking. Incorporating personal and homelike elements that address older adults’ aesthetic, social, and existential needs can soften clinical atmospheres, transforming corridors into meaningful parts of the living environment.

## Introduction

The International Classification of Functioning, Disability and Health (ICF) highlights the close interrelationship between health, functioning, and environmental factors (World Health Organization, 2018). Increasing evidence also links the quality of care to the design of the built care environment (Centre for Healthcare Architecture, 2020; Pickens et al., 2024). In Sweden, a national architectural policy promotes the development of supportive care environments as integral parts of designed living spaces (Boverket, 2022). Against this backdrop, the present study focuses on Swedish residential care facilities providing 24-h access to direct care staff, addressing both the care and housing needs of older adults aged 65 years and older. The study specifically aimed to explore meanings embedded in corridors and how these relate to older adults’ experiences of physical movement and their living environment within a residential care facility.

Aging involves not only changes in physical capacity but also shifts in how environments are perceived and experienced over time. Older adults’ interactions with the built environment are shaped by both functional needs and embodied experiences of movement, belonging, and agency (Wahl et al., 2012). In residential care settings, a homelike atmosphere and a sense of place have been described as important (Johansson et al., 2022), with design factors such as social space design, unit layout and size, sensory qualities, and homelike features influencing behavior and well-being (Chaudhury et al., 2018). Communal areas play a central role in social interaction, engagement in everyday activities, and overall well-being (Ribbe Kelso et al., 2024). At the same time, the physical environment can both constrain action through the affordances it offers, i.e., the possibilities for action available to a person based on their abilities and intentions (Gibson, 2014). In this sense, movement in later life is not only a matter of physical capacity but also an embodied and perceptual experience through which older adults relate to their environment.

*Movement in later life is not only a matter of physical capacity but also an embodied and perceptual experience through which older adults relate to their environment.*

Corridors in healthcare environments, although primarily designed for transport and circulation, are increasingly recognized as meaningful spaces that can shape care quality, social interaction, and individual experience (Colley et al., 2018; Jiang & Verderber, 2017). In residential care settings, corridor design has been shown to influence older adults’ energy consumption as well as their perceptions of comfort, orientation, and satisfaction (Hou et al., 2023). Despite this, corridor spaces remain largely understudied. A recent Cochrane review identified only one out of 20 studies that specifically addressed corridor spaces; most studies instead compared home-like and institutional care models, with inconclusive findings regarding quality of life, behavior, depression, daily activities, and adverse events (Harrison et al., 2022). In Sweden, Andersson (2011) found that many residential care facilities have a “hospital-like” design, with prominent corridors and multi-purpose spaces that prioritize care delivery over homelike qualities (Andersson, 2011). As many of these facilities are still in use, further research is needed to understand how older adults experience communal spaces such as corridors, which are central to everyday movement yet have received limited empirical attention.

Corridors can both support and limit interaction with the environment (Gibson, 2014), raising what Blundell-Jones and Meagher (2015) describe as “the problem of the corridor”: how such spaces can become more than mere circulation routes (Blundell-Jones & Meagher, 2015). Architectural solutions, such as incorporating activity spaces, seating niches, access to natural light, and clear visual cues, have shown potential to enhance corridor usability and autonomy (Colley et al., 2018; Hou et al., 2023). In line with person-centered care principles, care environments should support privacy, mobility, social interaction, and a sense of everyday life (Edvardsson, 2008; Nordin, 2016).

## Methods

### Theoretical Framing and Design

The study employed an explorative qualitative design using phenomenological hermeneutics as developed by Lindseth and Norberg (2004, 2022). The method is grounded in Ricoeur's (1976) theory of interpretation and seeks to illuminate meanings of lived experience through a dialectical process of understanding and explanation. It emphasizes understanding our world through history and narratives, linking the present with the past and the actual with the potential. To ensure transparent and comprehensive reporting, the Consolidated Criteria for Reporting Qualitative Research (COREQ, Tong et al., 2007) were followed (Supplementary Material 1).

### Pre-Understanding

In phenomenological-hermeneutic research, the researchers’ pre-understanding forms an interpretive backdrop that cannot be bracketed but must be acknowledged with critical reflection. The first author has several years of experience as a physiotherapist and a background in art and design. She currently works with organizational development at management level, focusing on residential care environments. The last author has extensive experience as an occupational therapist in aged care and now works as a senior lecturer and researcher in ageing and health. Their combined pre-understanding contributed sensitivity to embodied movement, spatial experience, and everyday life in residential care while also necessitating reflexive engagement throughout the study.

### Ethical Considerations

This study did not involve any procedures or interventions with research participants as defined in the Swedish Ethical Review Act and did thus not fall under the scope of mandatory ethical review. The Swedish Ethical Review Authority issued an advisory opinion for the study (dnr. 2022-04629-01), stating that they had no ethical objections to the study. All participants provided written informed consent to participating in the study, and all data are handled in accordance with European General Data Protection Regulation (GDPR EU 2016/679). Recorded data have been transcribed without mentioning people by name and all data are de-identified for publication.

### Context, Setting, and Participants

**Figure 1.**
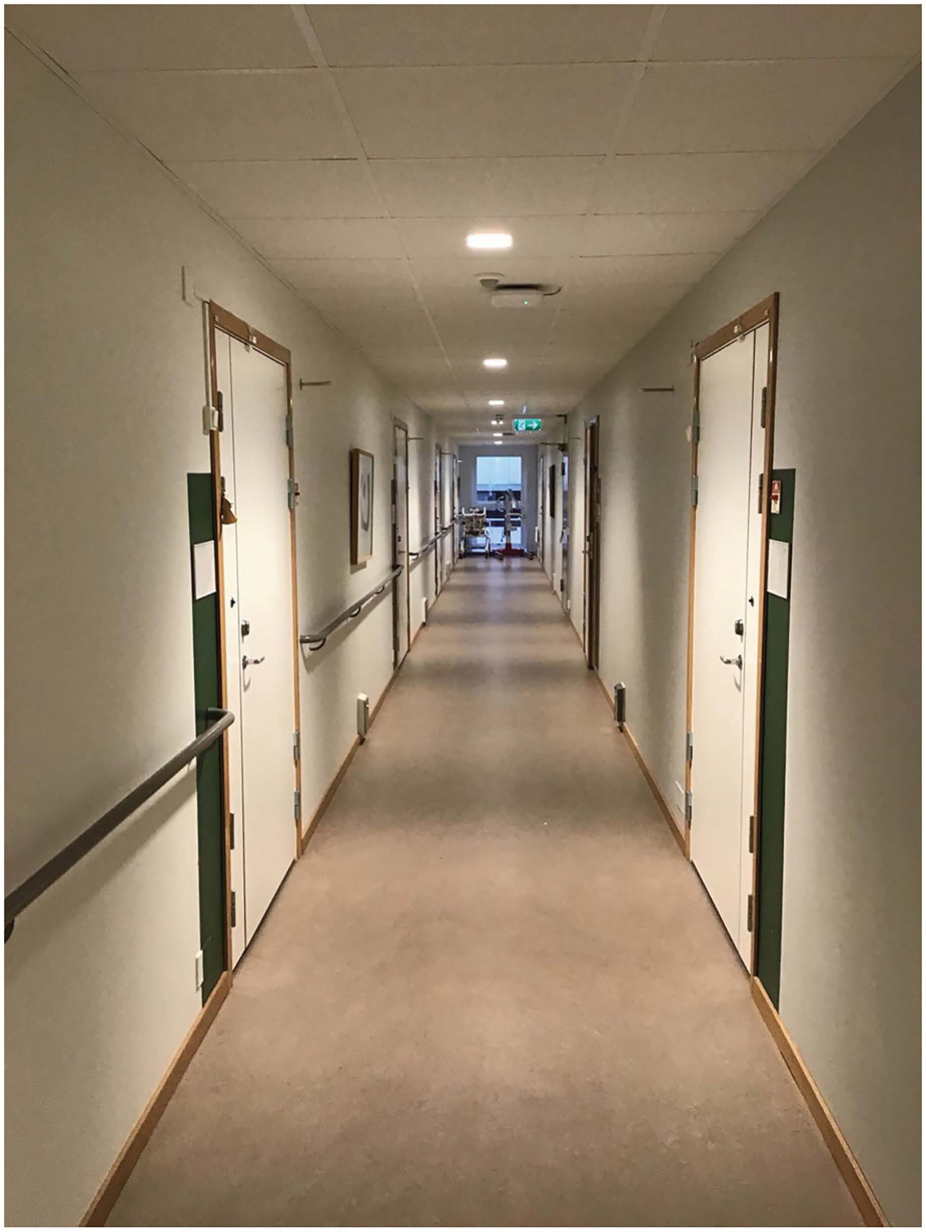
Photo from one of the corridors. All three corridors were identical.

The study was conducted in a single multi-story residential care facility built in the 1970s in a mid-sized Swedish city, comprising three units for persons with somatic disabilities. Focusing on one facility enabled in-depth exploration of older adults’ lived experiences within a shared and consistent physical environment, thereby supporting analytic depth, while minimizing variability related to architectural differences. All units shared an identical corridor layout: straight, brightly lit corridors approximately 48 m (157 ft) long and 1.6 m (5 ft and 3 inches) wide, with 16 visible doors, including apartment entrances, staff rooms, laundry rooms, an emergency staircase, and openings to shared spaces such as a coffee-room, dining room, and entryway (see Figure 1). The corridors had white walls and doors, brown wood skirting boards and casings, light grey-brown flooring and grey handrails along one side. Each apartment door was marked with a blue or green panel. One end of the corridor faced a window overlooking a residential building, while the other opened toward the main entrance, kitchen, and dining area. Surfaces and doors had been renovated one year prior to data generation, and residents’ nameplates were added during the study period.

Participants were purposively selected based on the following criteria: residence in the facility, ability to ambulate independently with or without walking aids or a wheelchair, or with support from no more than one person, and absence of diagnosed dementia (due to the spatial and cognitive demands of the interviews). Facility managers and staff identified eligible persons and provided study information, aiming for gender balance. Seven persons expressed interest; six (three men, three women), aged 81–96 years, consented to participate. Four used walking aids and two were independent wheelchair users capable of walking short distances. All six participants took part in one interview, and four completed a follow-up interview, resulting in a total of 10 interviews. The follow-up interviews enabled deeper exploration of participants’ experiences. Guided by the concept of information power (Malterud et al., 2016), the sample was considered sufficient given the study's focused aim, the specificity of the sample, and the depth of the interviews.

### Data Generation

Data were generated in Swedish through open, co-creative dialogues, conducted by the first author in a collaborative and iterative process, taking place partly while walking through the corridors to situate descriptions within the physical environment of interest. This approach allowed participants to evoke momentary, embodied, and situational experiences that may be difficult to recollect in seated interviews. All interviews followed an interview guide (Table 1) to ensure methodological coherence while allowing participants to shape the direction of the conversation. Participants determined the route and pace through the corridors, an approach comparable to “go-along” interviewing (Kusenbach, 2018). Initial interviews lasted 32–55 min, and follow-up interviews 47–83 min. Follow-up interviews offered opportunities to revisit and deepen earlier descriptions, serving both as an initial interpretive encounter and as a form of member checking to support trustworthiness. All interviews were digitally audio-recorded and transcribed verbatim by the first author. Manual fieldnotes, including sketches, provided contextualization of spatial features and participants’ movements.

**Table 1.** Interview Guide.

	Prompt/Question	Reminder to Author
Introduction/start in secluded area (apartment)	Invite the participant to share initial thoughts, e.g., asking if the research subject has any thoughts about the topic, about corridors or about moving through corridors—or their specific experiences of these particular corridors.	Prepare the participant to lead the tour in the corridors.
Walk/tour through corridors	Repeat words or phrases and ask the person to elaborate. Areas to probe when mentioned by participant: How the participant experiences the corridors, what is noticed How it feels in the body How the corridor affects the experience of body and movement Whether there is a difference in the experience from different places within the corridors or depending on how you move and how that difference is manifested	Let the participant lead. Experiences here and now—the person gets to lead the tour and talk freely about what they experience now when they move in these particular corridors. Try to reformulate and make initial interpretations of what is said, to increase understanding and consensus and deepen the conversation, as well as to validate/reject possible interpretations together with the participant.
Summary/conclusion in secluded area (apartment)	Ask participants to summarize experiences Ask if there is anything they want to tell that they did not feel they wanted to talk about in the corridors. In-depth and follow-up questions as described above. Invite participants to share concluding thoughts, e.g., new thoughts on the subject.	Summarize and make initial interpretations of what has been said during the interview, to validate/reject possible interpretations together with participant Thank participant

### Data Analysis

Interview data and fieldnotes were analyzed using Lindseth and Norberg's (2004, 2022) three stages: naïve understanding, structural analysis, and comprehensive understanding. These stages involve an iterative process of reading, writing, and shifting between the whole and its parts to interpret meaning. After transcription, both authors conducted a naïve reading of each interview to formulate a joint naïve understanding of the text as a whole. This shared tentative interpretation guided the subsequent structural analysis, which involved examinations of transcripts and fieldnotes to identify meaning units relevant to the study aim and the naïve understanding, condensing them to capture their essential meaning. These units were compared, clustered, and gradually developed into themes. Naming themes was a key interpretive step that required ongoing movement between parts and whole, between explanation and understanding. Emerging themes were discussed and refined by the authors. For an example of the interpretation process (see Table 2). Finally, a comprehensive understanding was developed by bringing together the naïve understanding, the structural analysis, and relevant theoretical perspectives. Themes were contextualized within relevant theory through critical reflection of the authors’ pre-understanding. To preserve linguistic nuance, the analysis was conducted in Swedish until the final stage, after which results were translated into English for reporting.

**Table 2.** Examples from the Interpretation Process.

Meaning Unit	Sorting Per Interpretive Question Based on Naïve Understanding	Condensed Unit	Example of Preliminary Sub-Theme During Further Interpretation	Final Theme
You don't walk as much as you should just because it's far (Participant 1, interview 2)	Question 1: What meaning is embedded in corridors relating to terms for movement, activities, or orientation?	The length of the corridor is perceived as physically demanding and thus restrictive, you walk less often	Physical spatial design and the activities of others are relevant for mobility	Movement shaped by the corridors’ materiality
“… they are long and boring (…) There are a few small paintings here and there, but it's like nothing catches the eye, something else, it's just the floor and all the doors.” (Participant 3, interview 1)	Question 2: What meaning is embedded in corridors relating to bodily, sensory and/or emotional experiences?	The corridor is perceived as boring because it is lacking content, nothing catches the eye	Discomfort due to lack of content, life versus death, emptiness, anonymity and nothingness in décor and design	Emotional atmospheres of the corridors
“It's pretty good to have [one's apartment] at the beginning of the corridor (…) But well, I guess that becomes a habit too, because you are forced to get into a new habit and live a new life. There is no choice.” (Participant 2, interview 1)	Question 3: What meaning is embedded in corridors relating to the overall experience of the living environment and life terms in a residential facility?	It would be more difficult and sad to live further down the corridor, but you would get used to it because you have to	You get used to it, accept your situation	Adapting to the corridors as adapting to a new life

### Naïve Understanding

The corridors are a transport route to the destination where you are going. How rooms are designed and how rooms are located in relation to each other is important for well-being and for what opportunities for movement there are. Adapted level of stimulation, variation in the indoor environment and social interaction are important, as well as contact with the outdoor environment.

The dimensions of the corridors are perceived to be obstacles both physically and psychologically and can cause discomfort. The corridors feel monotonous to move in, as empty and lifeless. They are impersonal and it is hard to find your way. The corridors can also provide a feeling/bodily experience of space.

Moving around in the corridors is a significant part of the participants’ overall experience of living in a residential care facility, and they are something you need to find an approach to and get used to. The corridors are not perceived as a part of/perceived to be outside one's home. The corridors have aspects that are appreciated, but the participants do not feel that the living environment has been created with their needs as whole people in mind. At the residential care facility, you are directed. The corridors make the residential care facility feel like an institution.

## Results

**Figure 2.**
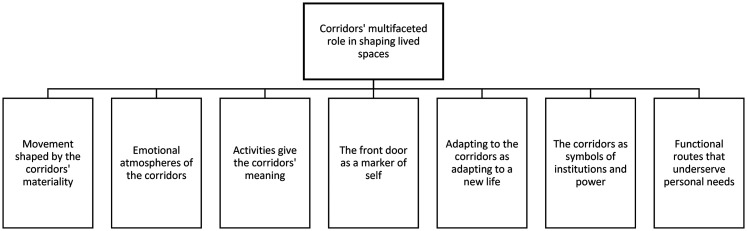
Overview of the thematic structure.

The interpretive analysis led to the overarching theme *Corridors’ multifaceted role in shaping lived spaces*, meaning that corridors were both architectural connectors and meaning-laden environments that shaped how participants moved, acted, and understood themselves within the facility. Meanings were embedded in everyday movement, influencing how participants navigated bodily limitations, sought or avoided social contact, maintained identity, and negotiated institutional constraints. Movement was never only functional but carried existential, emotional, and relational significance. Through corridor experiences, identities were affirmed or challenged and independence and belonging either supported or constrained. These meanings are elaborated in seven sub-themes below (see Figure 2).

### Sub-Theme 1: Movement Shaped by the Corridors’ Materiality

The corridors’ material form set the basic conditions for movement while simultaneously exposing participants’ physical vulnerabilities. Light, surfaces, visibility, and handrails provided reassurance and orientation, enabling movement that might otherwise feel unsafe. Yet the corridors’ narrowness and length accentuated physical strain, fatigue, and pain, turning a seemingly neutral space into an embodied challenge. Participants developed strategies to navigate tight spaces, illustrating how corridors both enabled and disciplined movement. Descriptions from two participants capture this duality, inviting movement through clarity while also questioning the body's capacity (Participant 1 [P1], Interview 2 [Int. 2]; Participant 2 [P2], Interview 1 [Int. 1]).

“It's a corridor that's straight to the elevator, for example. That’s appropriate.” (P2, Int. 1)

“If it had been shorter, one would have walked a little more often.” (P5, Int. 1)

### Sub-Theme 2: Emotional Atmospheres of the Corridors

Participants described the corridor as carrying emotional atmospheres that shaped their willingness to use it. Home-like elements, such as plants, warm colors, and pleasant scents, evoked comfort and attachment, making the corridor feel “alive” and inviting movement toward shared spaces. In contrast, darker or colder areas created unease, detachment, or avoidance, showing how affective tones can support or inhibit engagement. Participants spoke of being “drawn” toward pleasant areas such as the dining room end, while the opposite end felt “far away,” illustrating how emotional landscapes are mapped onto physical space. Two participant quotes reflect the deeper meanings embedded in these contrasting atmospheres:

“That small color field does a lot for me. Takes a place in one's heart a little more, and I feel for the room a little more. I don't really understand it myself but … I think it's nice.” (P2, Int. 1)

“The pressure is on you when you get over there, you know. It's dark and grey and boring. (…) Depressing in feeling I think. Confinement.” (P1, Int. 2)

### Sub-Theme 3: Activities Give the Corridors’ Meaning

Movement in the corridor was rarely aimless; participants moved with purpose toward activities that mattered to them. They also felt dependent on facility's range of available activities. Meals, social interaction, entertainment, and going outdoors gave direction and meaning, turning the corridors into pathways toward connection. When such activities were absent or inaccessible due to health or scheduling, participants described losing not only spatial orientation but also existential direction, as the corridor no longer offered a reason to move. A quote from participant three shows how meals could “pull” them through the corridor, illustrating how activities functioned as motivational anchors:

“You take a tour every now and then. We eat at least three times a day, so …” (P3, Int. 1)

### Sub-Theme 4: The Front Door as a Marker of Self

Participants’ apartment doors played a central role in preserving identity and self-determination. While the corridors signified shared life, the door marked personal territory and control. Nameplates and colored panels signaled a sense of “mine,” reducing confusion and protecting privacy. In an otherwise uniform corridor, the door became an assertion of personality and a tangible boundary between self and others, allowing participants to decide when to be alone and when to invite others in. Feelings of unsettlement when passing identical doors without clear identifiers revealed how easily spatial anonymity can disrupt personhood. A quote from participant three illustrates the door's significance:

“You go in and shut the door, and you're home. Then it's like a life-space, because here I can do and live how I … as I like it. Out there, there it is … You just have to take it as it comes. And not get into anyone's way.” (P3, Int. 2)

### Sub-Theme 5: Adapting to the Corridors as Adapting to a New Life

Becoming acquainted with the corridor was part of participants’ broader adaptation to life in the facility. Early experiences of navigating a long, shared institutional space, noticeably different from a private home, involved adjusting to walking through multiple doors to reach dining areas or the outdoors. Over time, familiarity with the layout and routines enabled a tentative sense of “at-homeness.” However, this adaptation carried emotional weight, involving an inner negotiation between accepting their new life situation and longing for a former home. A quote from participant two illustrates this gradual adjustment and the existential labor of reconciling loss, dependency, and search for normalcy:

“Now, I have grown into this, to me it’s … normal … (…) But if you can't grow into it, what then? Then your soul changes.” (P2, Int 1.)

### Sub-Theme 6: The Corridors as Symbols of Institutions and Power

For several participants, the corridors symbolized more than its physical form; it evoked memories of hospitals, schools, or even prisons, places associated with authority, surveillance, and limited choice and control. These associations illuminated the power relations embedded in the environment. Daily movement through the corridors reminded participants of the facility's institutional nature, at times generating feelings of alienation and being directed, with personal needs overshadowed by organizational routines. Participants acknowledge the challenge of accommodating diverse resident needs, noting how the architecture embodied institutional logic while still providing necessary support when independent living was no longer possible. A quote from participant one illustrates the experience of corridors as a symbol of institutions and power:

“… You feel a bit alienated. (…) It feels a little more institutionalized than you would like. (…) It's the length of the corridors and all the doors and things like that. The sameness like, it's like … Nothing personal about it. It feels like it's more institutional.” (P1, Int. 1)

### Subtheme 7: Functional Routes That Underserve Personal Needs

While crucial as transport routes, the corridors were often experienced as inadequate for supporting social, emotional, and existential needs. Their linear, repetitive design encouraged swift movement rather than lingering, limiting spontaneity, interaction, and engagement. Participants expressed a desire for greater variation, opportunities for interaction, and activity cues that could make the corridor feel “alive” rather than monotonous and invite time spent outside their apartments. In the absence of such stimulation and human presence, corridors were experienced as “dead”, reinforcing feelings of invisibility and disconnection, as illustrated by the following quote from participant two:

“Life here in a residential care facility is monotonous and one-sided, and it doesn’t get any better with a long corridor that never ends. One thing builds on the other.” (P2, Int 1.)

### Comprehensive Understanding

Taken together, the themes illustrate the multifaceted and sometimes contradictory meanings that corridor spaces hold in older adults’ everyday lives. Participants encountered and negotiated the corridors through their bodies, emotions, identities, and relationships with others and the institutional environment. These interpretations point toward a deeper, integrated understanding of corridors as lived spaces. In line with the final interpretive movement in phenomenological-hermeneutic analysis (Lindseth & Norberg, 2004), this comprehensive understanding weaves together the naïve understanding, structural analyses, and theoretical perspectives through the theoretical lenses of sociomateriality (Gherardi, 2016a, 2016b), place/placelessness (Relph, 1976; Seamon, 2023), and affordances (Gibson, 2014). These perspectives illustrate how the meanings of corridors are interconnected and reflect the complexity of older adults’ life-worlds. These perspectives underscore the interplay between material, social, and symbolic dimensions, the importance of a sense of place, and the existential consequences of placelessness. This aligns with Ricoeur's (1976) notion of interpretation as opening new possibilities for understanding the world.

*Participants encountered and negotiated the corridors through their bodies, emotions, identities, and relationships with others and the institutional environment.*

Sociomateriality defines how meaning arises through interplay between bodies, objects, routines, and environments (Gherardi, 2016a, 2016b). Participants’ movements, interactions with building features, use of walking aids, and daily routines exemplify such sociomaterial entanglements. From the perspective of phenomenology of place (Seamon, 2023), participants’ accounts emphasize their immersion in specific spatial, social, historical, and existential contexts. Experiences of placelessness, described by Relph (1976) as a negative existential experience arising from the loss of unique and varied experiences and identities of places, emerged where environments failed to support identity or belonging. Affordance theory (Gibson, 2014) further clarifies how corridor dimensions, features, and available activities either support or constrain older adults’ ability to move, interact, and feel at home, especially for those with physical impairments. Accordingly, the meanings of corridors involve access to environments that support movement, identity, connection, and the freedom to engage with these spaces in ways that affirm autonomy, sense of place, and well-being.

## Discussion

Previous research shows that older adults in residential care seek a sense of place through their interactions with the environment, supported by engaging in social activities, expressing personal identity, and building interpersonal relationships (Falk et al., 2012). Johansson et al. (2022) further argue communal areas should be designed holistically to support residents’ desires, rather than relying on isolated environmental features (Johansson et al., 2022). The present study extends this work by illustrating how corridors can either support or hinder movement, interaction, and personal expression. Corridors that enable meaningful engagement and continuity of self, can contribute to a stronger sense of place, whereas those that restrict agency risk undermining well-being.

*Corridors that enable meaningful engagement and continuity of self, can contribute to a stronger sense of place, whereas those that restrict agency risk undermining well-being.*

Homelikeness is often presented as the opposite of institutionalization (Johansson et al., 2022), yet participants’ experiences revealed how corridors symbolized institutional control and power. Maintaining identity and agency in this context required both environmental and interpersonal support. Similar concerns about institutional cultures in residential care have been raised in previous research (Hermansen Østby et al., 2025), emphasizing that staff play a crucial mediating role between organizational demands and residents’ needs. Our findings align with this perspective, reinforcing the need for environments and practices that accommodate diverse and changing needs among older adults.

Person-centered care is widely recognized as fundamental to high-quality residential care (Ballard et al., 2018), with documented benefits for well-being and neuropsychiatric symptoms (Kim & Park, 2017). Achieving a person-centered approach requires attention not only to care practices but also to the physical care environment and organizational structures that shape everyday life in residential settings (Mohr et al., 2021). A person-centered climate is characterized by safety, everydayness, and hospitality, qualities that encompass both physical and psychosocial aspects of the environment (Edvardsson et al., 2008). Enriching corridors and other communal areas to reflect these characteristics may strengthen person-centered care by supporting familiar, welcoming, and socially supportive environments. In line with recent empirical work, our findings suggest that corridor design should address both physical and emotional wellbeing, as integrating opportunities for meaningful activity, social interaction, and recognition may support older adults in feeling acknowledged, supported and at home. At the same time, the corridors in this study did not fully support person-centered care. Their institutional character and limited affordances constrained opportunities for interaction, autonomy, and everydayness. Research on supportive environments (McGann et al., 2024) emphasizes the importance of homelike qualities and building typologies that facilitate meaningful engagement, while also highlighting the need for more interview-based studies that foreground older adults’ own perspectives (McGann et al., 2024). This study contributes to that body of literature by illustrating how corridor design influences everyday movement, identity, and well-being from older adults’ own point of view.

*Our findings suggest that corridor design should address both physical and emotional wellbeing, as integrating opportunities for meaningful activity, social interaction, and recognition may support older adults in feeling acknowledged, supported and at home.*

### Methodological Considerations

As a small exploratory study, recruitment was facilitated by staff and managers and limited to physically active older adults without diagnosed cognitive impairment, which likely influenced the results, as participants were healthier than many residents in Swedish residential care. Given the study context, this was the most feasible and ethically appropriate approach. In phenomenological hermeneutic research, validity rests on the interplay between understanding and explanation. In this study, this was embedded throughout the research process through co-creative dialogue, iterative interpretation, and follow-up interviews that allowed emerging meanings to be revisited and nuanced. Rather than treating member checking as a separate step, validation occurred through the dialogical interview process. Credibility was further strengthened through joint analytical discussions among the authors, with interpretations continuously assessed against the naïve understanding and empirical data.

## Conclusion

This study shows that corridors in residential care facilities are more than functional passageways; they are lived spaces that shape older adults’ movement, identity, and well-being. The findings illustrate how material, social, and symbolic dimensions of corridors intertwine to support or undermine sense of place, self-determination, and connection. This underscores the need to extend person-centeredness beyond care practices to the architectural and organizational contexts that shape everyday life. By highlighting corridors as spaces of both possibility and vulnerability, the study emphasizes the importance of environments respond to older adults’ aesthetic, social, and existential experiences. Attending to these qualities, rather than focusing solely on technical or efficiency-driven concerns, may transform corridors into supportive and life-enhancing parts of the living environment, strengthening personhood and meaningful engagement in everyday life.

## Implications for Practice

- The complexity of corridor spaces places demands on architects, designers, facility staff and managers to understand, clarify, and respond to older adults’ lived experiences.
- Corridor spaces should be recognized as integral parts of older adults’ everyday living environments in residential care facilities.
- The design of corridor spaces in residential care should address aesthetic, social, and existential aspects of older adults’ health.
- Power dynamics in residential care environments require design and refurbishment processes that actively involve and empower older adults.

## Supplemental Material

sj-pdf-1-her-10.1177_19375867261431866 - Supplemental material for The Multifaceted Role of Corridors in Residential Care Facilities: A Phenomenological Hermeneutic Study With Older Adults Supplemental material, sj-pdf-1-her-10.1177_19375867261431866 for The Multifaceted Role of Corridors in Residential Care Facilities: A Phenomenological Hermeneutic Study With Older Adults by Karin Elias and Qarin Lood in HERD: Health Environments Research & Design Journal
